# Expression of *hsa-MIR-204, RUNX2, PPARγ,* and *BCL2* in Bone Marrow Derived Mesenchymal Stem Cells from Multiple Myeloma Patients and Normal Individuals

**DOI:** 10.22074/cellj.2017.4480

**Published:** 2017-05-17

**Authors:** Raziyeh Mansurabadi, Saeid Abroun, Abass Hajifathali, Amir Asri, Amir Atashi, Mansoureh Haghighi

**Affiliations:** 1 Department of Hematology, Faculty of Medical Sciences, Tarbiat Modares University, Tehran, Iran; 2 Bone Marrow Transplantation Center, Taleghani Hospital, Shahid Beheshti University of Medical Sciences, Tehran, Iran; 3 Department of Clinical Biochemistry, Faculty of Pharmacy and Pharmaceutical Sciences, Isfahan University of Medical Sciences, Iran

**Keywords:** Multiple Myeloma, Mesenchymal Stem Cells, *hsa-MIR-204*, *RUNX2*

## Abstract

**Objective:**

Multiple Myeloma (MM) is a heterogeneous cytogenetic disorder in which clonal plasma cells proliferate in the bone marrow (BM) and cause bone destruction. The BM microenvironment plays a crucial role in pathogenesis of this disease, and mesenchymal stem cells (MSCs) are one of the key players. Herein, we propose to investigate the expressions of *hsa-MIR-204*, runt-related transcription factor 2 (*RUNX2*), peroxisome proliferator-activated receptor gamma (*PPARγ*), and B-cell lymphoma 2 (*BCL2*) as factors involved in osteogenesis, adipogenesis, and MSC survival in BM-MSCs from MM patients and normal individuals.

**Materials and Methods:**

In this experimental study, we isolated MSCs from BM aspirates of MM patients and healthy donors. Total RNA were extracted before and after co-culture with L363 myeloma cells. Gene expressions of *RUNX2*, *PPARγ*, *BCL2*, and *hsa-MIR-204* were assessed by quantitive real time polymerase chain reaction (qRT-PCR).

**Results:**

Higher levels of *RUNX2*, *PPARγ*, and *hsa-MIR-204* expressions existed in MM- MSCs compared to normally derived (ND)-MSCs. *BCL2* expression decreased in MM- MSCs. We observed different results in the co-culture model.

**Conclusion:**

In general, the MM-MSCs gene expression profile differed compared to ND- MSCs. Upregulation of *RUNX2*, *PPARγ*, and *hsa-MIR-204* in MM-MSCs compared to ND- MSCs would result in formation of bone defects. Downregulation of *BCL2* would lead to MM-MSC cell death.

## Introduction

Multiple myeloma (MM) is a cytogenetically heterogeneous disorder in which clonal plasma cells, as the main players, produce high levels of monoclonal immunoglobulins (1). Malignant plasma cells proliferate in bone marrow (BM), resulting in bone destruction (2). Lytic bone lesions arise in 90% of patients as the result of perturbations in bone remodeling homeostasis (3). The BM microenvironment in which MM develops plays a crucial role in retaining plasma cell growth, proliferation, and survival. The BM microenvironment in MM is formed of a threedimensional structure of sub-microenvironments that include osteoblasts and vascular niches, and is infiltrated by clones of plasma cells, extracellular matrix (ECM) proteins, and BM stromal cells (4). This neoplastic niche supports and maintains the development of tumor cells by interactions between multiple cell types and molecules (5).

Mesenchymal stem cells (MSCs), as stromal cells, have the capability to differentiate to different cell types including osteoblasts and adipocytes (6). Suppression of osteoblast differentiation and activity, along with increasing the apoptosis of osteoblast cells, are the most important causerelated bone destruction processes (7). The B-cell lymphoma 2 (*BCL2*) protein, as one of the antiapoptotic factors in a wide variety of human cell systems, plays a pivotal role in the apoptosis process (8, 9). Runt-related transcription factor 2 (*RUNX2*) is a Runt domain transcription factor that has an important role in activation of genes involved in osteoblast and chondrocyte differentiation, which is controlled by transcriptional and posttranscriptional mechanisms (10, 11). Peroxisome proliferator-activated receptor gamma (*PPARγ*), a member of the ligand-activated nuclear receptor superfamily of transcription factors, is an important adipogenic factor that increases cellular lipid levels and decreases bone formation (12, 13).

The involvement of microRNAs in pathogenesis of many cancers has been demonstrated (14). MicroRNAs are a class of endogenous short noncoding RNAs that regulate post-transcriptional gene expression by binding to 3ˊ untranslated regions (3ˊ UTRs) of target mRNAs (15, 16). They can be considered diagnostic and prognostic markers for cancer (14). Several experiments have shown the different profiles of microRNA expression in MM patients (17). MicroRNAs also participate in different cell fates, as it has been shown that they interfere with osteoblast differentiation in either a positive or negative manner (18). MiR-204 and its homologue miR- 211 express in different mesenchymal progenitor cell lines and BMSCs. During adipogenesis, the expressions of these molecules upregulate (12). In the present study we proposed to investigate *hsa-MIR-204*, *RUNX2*, *BCL2*, and *PPARγ* gene expressions in MM-MSCs compared to normally derived (ND)-MSCs.

## Materials and Methods

### Bone marrow mesenchymal stem cell isolation and culture

In this experimental study, BM aspirates from 4 male MM patients that ranged in age from 50-70 years and 2 healthy donors, matched for age and sex, were obtained by surgeons at Taleghani and Imam Khomeini Hospitals (Tehran, Iran). We included MM patients in this study after verification of their disease by pathology reports and BM aspirate smears. MM patients underwent no chemotherapy, radiotherapy, or surgery. Healthy donors volunteered their BM and had no histories of cancer or autoimmune diseases. All samples were obtained after informed consent and in accordance with the TMU Ethics Committee (Reference number: D5505/52). Briefly, BM aspirates were diluted with phosphate-buffered saline (PBS, Sigma, USA) after which mononuclear cells were isolated by Ficoll density gradient centrifugation (GE Healthcare Life Sciences). Mononuclear cells were then washed with PBS and cultured in Dulbecco’s modified Eagle’s medium (DMEM, Gibco, USA) supplemented with 10% fetal bovine serum (Gibco, USA) and 1% penicillin/streptomycin (Sigma- Aldrich, USA). Next the cells were incubated in a humidified environment at a temperature of 37˚C and 5% CO2. After 48 hours, we removed any nonadherent cells and other debris, and cultured the MSCs. This study used only passage-4 MSCs. We divided the MM-MSCs according to the percentage of plasma cells that infiltrated into the BM as follows: 30% BM plasma cells (BMPCs), 40% BMPCs, and 70% BMPCs. We purchased the L363 myeloma cell line from Pasteur Institute of Iran. The cells were cultured in RPMI1640 (Gibco, USA) medium supplemented with 10% fetal bovine saline (FBS) and 1% penicillin/streptomycin.

### Flow cytometry analysis of bone marrow mesenchymal stem cells

We characterized the BM-MSCs according to immunophenotype by labeling the cells with human anti-CD105 conjugated to fluorescein isothiocyanate (FITC, eBioscience, USA), anti-CD90 conjugated to phycoerythrin (PE, eBioscience), and anti-CD45 FITC (eBioscience, USA). The cells were analyzed by FACsCalibur (BD Biosciences, USA).

### Differentiation of bone marrow mesenchymal stem cells to osteocytes and adipocytes

The BM-MSCs were plated in 12-well plates at 4×104 cells/well and cultured overnight to achieve adherence. We removed the medium and added differentiation media. In order to establish osteoblast differentiation, we cultured the BM-MSCs for up to 14 days in the presence of growth medium that contained 50 μg/ml of ascorbic acid, 10 mM β-glycerophosphate, and 10 nM dexamethasone, after which they were stained with alizarin red. For adipogenic differentiation, we cultured the MSCs up to 14 days in adipocyte-inducing medium that contained 1 μM dexamethasone, 0.5 mM methyl isobutyl xanthine, 10 μg/ml of insulin, and 100 μM indomethacin, after which the cells were stained with oil red O.

### Bone marrow mesenchymal stem cell co-culture with the L363 cell line

We plated the BM-MSCs in 6-well plates (6×104 cells/well). After 24-hour incubation in DMEM medium, the cells were washed with PBS to remove any non-viable and non-adherent cells. Then, 6×104 cells/well of L363 cells were co-cultured with direct cell-to-cell contact with the BM-MSCs. The medium was changed with an equal amount of DMEM and RPMI1640 medium for up to 48 hours, after which the suspension of L363 cells was washed with PBS and we harvested the adherent MSCs for molecular analysis.

### RNA extraction and cDNA synthesis

Total RNA was isolated using RNX-plus (Cinnagen, Iran) following the manufacturer’s instructions. RNA quality and concentration were determined after extraction using a biophotometer (Eppendorf, UK) and electrophoresis on 2% agarose gel. For cDNA synthesis, 2 μg of total RNA were reverse transcribed using a random hexamer primer and M-MuLV reverse transcriptase (Fermentas, USA) for 60 minutes at 42˚C.

### Quantitative real-time polymerase chain reaction

Briefly, 0.5 μl of cDNA was diluted in a total volume of 10 μl that contained 10 pmol of each of the primers and 5 μl SYBR Green Master Mix (Applied Biosystems, USA). Thermal cycling was initiated with denaturation at 95˚C for 10 minutes, followed by 40 cycles that consisted of denaturation at 95˚C for 10 seconds, annealing and extension at 60˚C for 60 seconds. Primers were obtained from SinaClon Company (Iran). The relative quantity of *RUNX2*, *BCL2*, and *PPARγ* gene expressions were normalized to GAPDH and *hsa-MIR-204* was normalized to SNORD expression to show absolute values of mRNAs or miRNA, respectively. Table 1 lists the sequences of primers used to quantify the desired genes.

**Table 1:** Primer sequences used for qRT-PCR


Gene	Primer sequences (5ˊ-3ˊ)

*hsa-MIR-204*	RT: GGTCGTATGCAGAGCAGGGTCCGAGGTATCCATCGCACGCAT CGCACTGCATACGACCAGGCATAG
	F: GCGATTCCCTTTGTCATC
	R: GAGCAGGGTCCGAGGT
*SNORD*	F: ATCACTGTAAAACCGTTCCA
*RUNX2*	F: GCCTTCAAGGTGGTA GCC C
	R: CGTTACCCGCCATGACAG TA
*PPARγ*	F: CCCTTCACTACTGTTGACTTC
	R: TCAGAATAATAAGGTGGAGATGC
*BCL2*	F: GTACTTAAAAAATACAACATCACAG
	R: CTTGATTCTGGTGTTTCCC
*GAPDH*	F: ATGGGGAAGGTGAAGGTCG
	R: TAAAAGCAGCCCTGGTGACC


RT; Reverse transcriptase and qRT-PCR; Quantitive real time polymerase chain reaction.

### Statistical analysis

The relative quantity of gene expression was analyzed using the 2^-ΔΔCT^ method. Differences between patients and control groups according to the Mann-Whitney and Kruskal-Wallis H nonparametric tests were considered significant at P<0.05. Graphs were designed by GraphPad Prism 5.

## Results

### Mesenchymal stem cells showed morphologic and phenotypic stem cell characteristics

**Fig.1:**
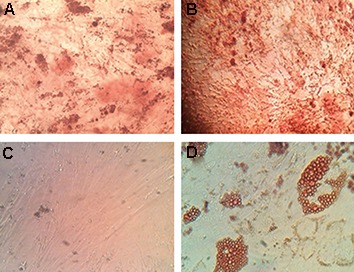
Mesenchymal stem cell (MSC) differentiation to osteocyte and adipocyte cell types after staining with oil red O for lipid and alizarin red for calcium deposition. A. Normal MSC osteocyte differentiation, B. Multiple myeloma (MM)-MSC osteocyte differentiation, C. Normal MSCs adipocyte differentiation, and D. MM-MSCs adipocyte differentiation (magnification: ×20).

**Fig.2:**
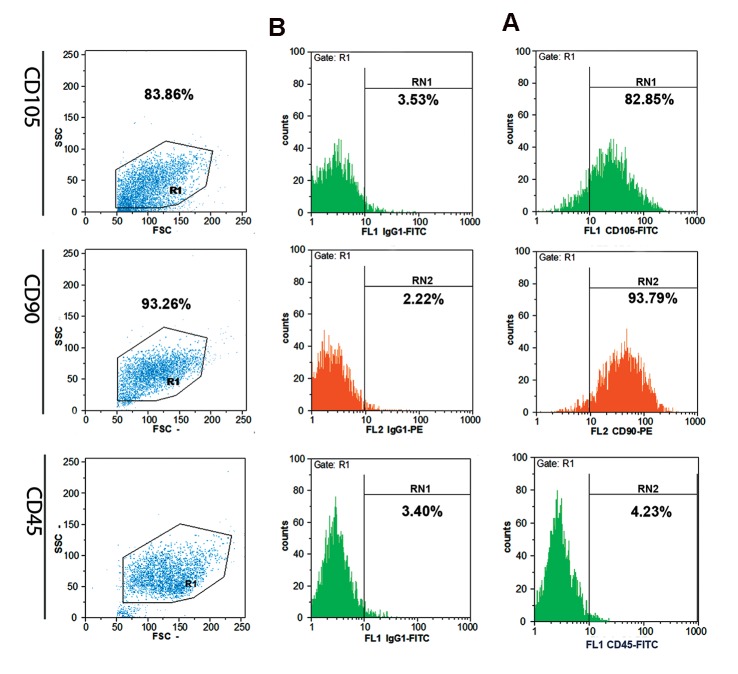
Schematic representation of flow cytometry analysis for expressions of CD105, CD90 and CD45 on MSCs. Column A; Labeled MSCs and Column B; Isotype controls.

The adherent MSCs had a spindle shape appearance. Figure 1 shows an example of *in vitro* differentiation of MM-MSCs and ND-MSCs into osteocytes and adipocytes. Images were captured at the end of 14 days culture under differentiation conditions. Alizarin red and Oil-red-O stains confirmed calcium deposition and accumulation of lipid vacuoles in the MSCs. Flow cytometry analysis showed that MM- and ND-MSCs both had 80% positivity for CD105 and both were 90% positive for CD90. They showed no significant expression of CD45 as a hematopoietic marker. The typical staining profile of the cells is presented in Figure 2.

### *RUNX2* and *PPARγ* expression in multiple myeloma-derived mesenchymal stem cells and normal derived-mesenchymal stem cells

**Fig.3:**
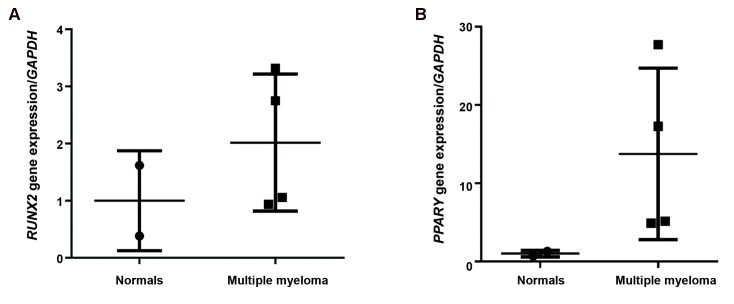
Results of quantitive real time polymerase chain reaction (qRT-PCR). A. *RUNX2* and B. *PPARγ* gene transcripts in multiple myeloma mesenchymal stem cells (MM-MSCs) compared with normal derived (ND)-MSCs. P<0.05 for both genes.

**Fig.4:**
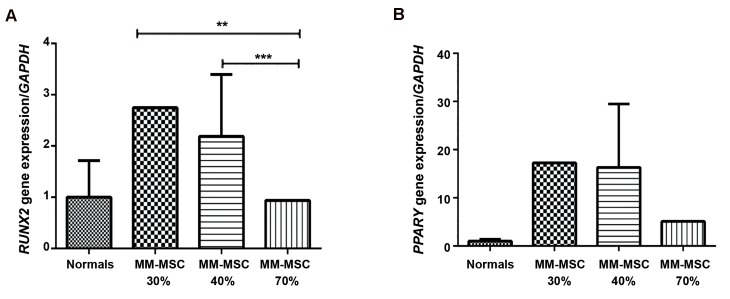
Results of quantitive real time polymerase chain reaction (qRT-PCR). A. *RUNX2* and B. *PPARγ* mRNA expression in multiple myeloma mesenchymal stem cells (MM-MSCs) derived from bone marrow (BM) aspirates infiltrated with 30, 40, and 70% plasma cells compared to normal derived (ND)-MSCs. **; P=0.004 and ***; P=0.0003.

We assessed for mRNA expressions of *RUNX2* and *PPARγ* in MM-MSCs and ND-MSCs. Results showed 2-fold greater *RUNX2* expression in MM-MSCs compared to ND-MSCs (Fig.3, P<0.05). We investigated the differences of these gene expressions in the 30, 40, and 70% MSC-BMPC groups. *RUNX2* expression decreased with increased percentages of BMPCs. The 30% BMPC group had 2.9-fold greater (P=0.002) *RUNX2* expression, and the 40% BMPC group expressed *RUNX2* 2.3-fold greater (P=0.0003) compared to the 70% BMPCs (Fig.4). *PPARγ* expression increased 12.8-fold in MM-MSCs compared to ND-MSCs (P<05, Fig.3) and showed a similar trend with *RUNX2* regarding the increased BMPCs. The 70% BMPCs had 3.3-fold lower *PPARγ* expression than the 30% BMPCs and 3.1-fold less than the 40% BMPCs (P<0.05, Fig.4).

### Comparison of *BCL2* expression in multiple myeloma-derived mesenchymal stem cells to normal derived-mesenchymal stem cells

**Fig.5:**
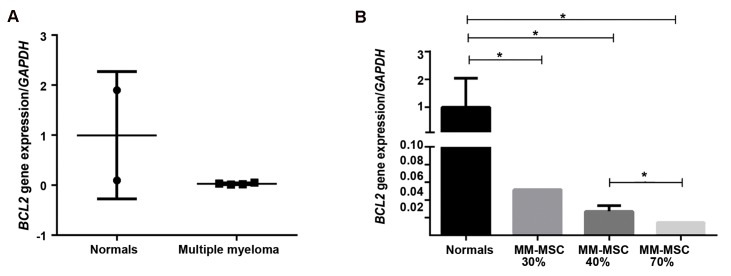
BCL-2 mRNA expression. A. multiple myeloma mesenchymal stem cells (MM-MSCs) and normal derived (ND)-MSCs and B. MM-MSCs derived from bone marrow (BM) aspirates infiltrated with 30, 40 and 70% plasma cells compared to ND-MSCs. *; P<0.05.

We assessed mRNA expression of *BCL2* in MM-MSCs and ND-MSCs as an additional indicator of MSC survival. As illustrated in Figure 5, *BCL2* expression decreased 33-fold in MM-MSCs compared to ND-MSCs (P<0.05). The 70% BMPCs showed 5-fold (P<0.05) decreased expression of *BCL2* compared to the 30% BMPCs and 2-fold lower than the 40% BMPCs (P=0.02).

### Comparison of *hsa-MIR-204* expression in multiple myeloma-derived mesenchymal stem cells with normal derived-mesenchymal stem cells

**Fig.6:**
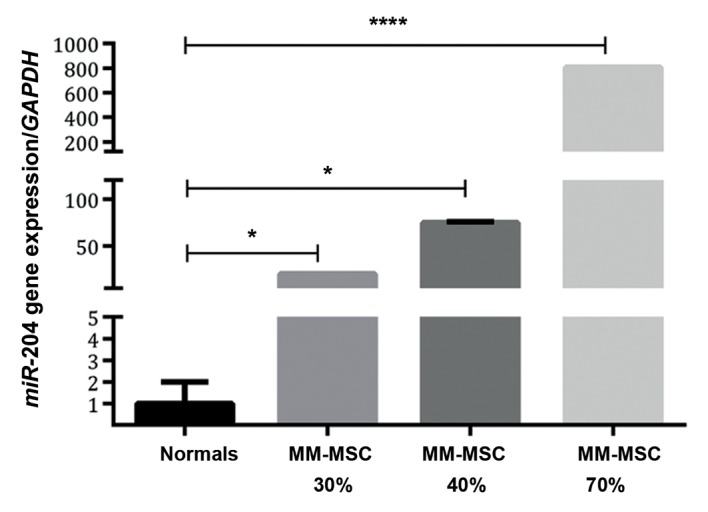
*hsa-MIR-204* expression in multiple myeloma mesenchymal stem cells (MM-MSCs) derived from bone marrow (BM) aspirates infiltrated with 30, 40, and 70% plasma cells compared to normal derived (ND)-MSCs. *; P<0.05 and ****; P=0.0003.

*hsa-MIR-204* expression in MM-MSCs was 244-fold higher than ND-MSCs (P<0.05). Increased percentages of BMPCs infiltration resulted in increased *hsa-MIR-204* expression. The 40% BMPCs expressed *hsa-MIR-204* 3.7- fold (P=0.02) greater than 30% BMPCs. The 70% BMPCs expressed *hsa-MIR-204* at a 10.7- fold (P=0.02) higher level than 40% BMPCs (Fig.6).

### Gene expression of *RUNX2*, *BCL2*, *PPARγ*, and *hsa-MIR-204* in bone marrow mesenchymal stem cells after co-culture with L363 myeloma cells

**Fig.7:**
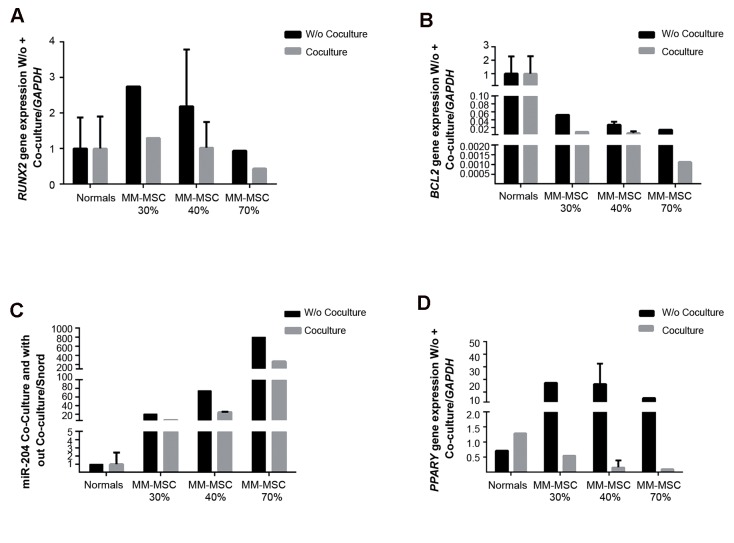
Results of quantitive real time polymerase chain reaction (qRT-PCR). Expression of A. *RUNX2*, B. BCL-2, C. *hsa-MIR-204*, and D. *PPARγ* in mesenchymal stem cells (MSCs) before and after co-culture with the L363 myeloma cell line.

We examined the effect of the L363 myeloma cell line on expression profiles of *RUNX2*, *BCL2*, *PPARγ*, and *hsa-MIR-204* in MSCs. Cells were co-cultured with L363 myeloma cells for 48 hours. The data showed that *RUNX2*, *BCL2*, *hsa-MIR-204*, and *PPARγ* expressions in MM-MSCs decreased following co-culture with L363 myeloma cells. ND-MSCs showed no significant differences in their gene expression profile after co-culture. The expression of *PPARγ* increased in ND-MSCs. The results demonstrated that *PPARγ* decreased in MM-MSCs after co-culture with L363, although ND-MSCs showed an increase in this gene after co-culture with myeloma cells (Fig.7).

## Discussion

Bone lesions are the most likely cause for disability and morbidity during the progression of MM. It is of interest to identify molecular pathways involved in this process as part of recent cancer based investigations. MSCs are capable of multilineage differentiation (adipogenesis and osteogenesis). These processes are regulated by several gene expressions such as *RUNX2* and *PPARγ* (19). It is well established that microRNAs interfere with the translational process in cells. Approximately one-third of the encoded genes are regulated by these molecules (20). *hsa-MIR-204* expresses in MSCs and regulates osteogenesis differentiation by targeting *RUNX2*, a key transcription factor of osteogenesis (12, 19, 21). Here, we have first compared the expression of *hsa-MIR-204* in NDMSCs and MM-MSCs. The data showed higher *hsa-MIR-204* expression in MM-MSCs compared to ND-MSCs, and a positive increased trend with increased BMPCs derived from MSCs. This data confirmed the results of other studies that MSCs abundantly expressed *hsa-MIR-204* (12, 22, 23). However we have demonstrated that MM-MSCs are more capable of expression. Next, we assessed the expression of *RUNX2* in MSCs. We observed higher expression of *RUNX2* in MM-MSCs (30 and 40% BMPCs) compared to ND-MSCs. MMMSCs derived from samples with 70% BMPCs had reduced *RUNX2* expression. Possibly, in the early disease stages the percentage of BMPCs was low and MM-MSCs compensationally expressed *RUNX2* to prevent bone lesions. However, progression of MM (70% BMPCs) caused decreased *RUNX2* expression, which was less than seen in ND-MSCs with progression of bone lesions. Simultaneous analysis of *RUNX2* and *hsa-MIR-204* indicated that this microRNA was a good regulator of *RUNX2*. Huang et al. (12) observed downregulation of *RUNX2* by *hsa-MIR-204* in mesenchymal progenitor cells. There was a negative effect on osteoblast differentiation by attenuation of *RUNX2* expression. Consistent with this study, we compared the expressions of *RUNX2* and hsa-MIR204 during disease progression. We observed that at a higher stage of MM (70% BMPCs), *hsa-MIR-204* had high expression and undoubtedly would have an eminent role in augmentation of bone lesions compared to a lesser disease stage (30 and 40% BMPCs) which showed decreased expression of *hsa-MIR-204* along with good expression of *RUNX2* to prevent bone disorders.

It has been reported that *PPARγ* acts as a *RUNX2* antagonist (19). Based on the decrease in *RUNX2* expression and defect in bone formation, expression of adipogenic factors would be expected to increase and osteoblast differentiation would switch to adipogenic differentiation (12). In line with these reports, our results have shown that *PPARγ* had higher expression in MM-MSCs from samples with 30 and 40% BMPCs compared to ND-MSCs. However we observed reduced expression in MM-MSCs from 70% BMPCs, which could be explained by cellular death at disease higher stages. Therefore, there were reduced numbers of expressed cells at the higher stages. Wang et al. (24) reported that *PPARγ* played a role in inhibition of the adhesive interaction between MM and BM. According to the higher expression of this molecule in MM-MSCs compared to ND-MSCs, possibly an increase of this gene in MM patients would have an important role in displacement of plasma cells from the BM niche and metastasis. This would require additional investigation.

Several studies demonstrated the effects of *hsa-MIR-204* on BCL-2 expression (9). Growth suppression of human hepatocellular carcinoma cells by *hsa-MIR-204* that targeted *BCL2* was reported by Li et al. (25). *hsa-MIR-204*, by targeting the 3´ un-translated region of *BCL2*, resulted in suppression of this gene expression (26). Sacconi et al. (9) observed that regulatory reduction of *hsa-MIR-204* as a prognostic factor caused increased *BCL2* expression. In the current study, we reported decreased expression of *BCL2* in MM-MSCs compared to ND-MSCs. As the percentages of PCMB increased, *BCL2* had more reduction which was in line with enhanced *hsa-MIR-204* expression. In terms of the important role of *BCL2* in cell survival, it is feasible that during disease progression, there is a decreased survival rate. It has been reported that *PPARγ*, as an E3 ubiquitin ligase, causes *BCL2* reduction (27). In the present study we identified increased *PPARγ* expression which contrasted *BCL2* expression.

Due to the probable interaction of MSCs with tumor cells in the BM niche in MM patients (28), we proposed to investigate the gene expressions after co-culture of MSCs with L363 myeloma cells. The results revealed that expressions of **hsa-MIR-204*, *RUNX2*, *PPARγ*,* and **BCL2** decreased in MM-MSCs following co-culture with L363 myeloma cells. Interaction of MSCs with myeloma cells appeared to reduce expression of *BCL2*, which led to cell death and resulted in low expressions of other genes. Possibly, with increased tumor cells, the expression of *RUNX2* reduced and inhibited bone formation.

## Conclusion

Collectively, for the first time, we showed that *hsa-MIR-204* had higher expression in MM-MSCs compared to ND-MSCs. The gene expression of *BCL2* as a survival factor and *RUNX2* as an osteogenesis factor decreased in MM-MSCs (70% BMPCs) compared with ND-MSCs, which could result in bone lesion progression in MM patients. We showed that *PPARγ* expression in MM-MSCs increased, which caused adipogenesis differentiation. These data would have beneficial approaches in designing appropriate therapy for MM patients. Relevant research with more samples would be required for more confirmation.
